# Middle Meningeal Artery Embolization for Chronic Subdural Hematoma

**DOI:** 10.3389/fneur.2020.557233

**Published:** 2020-10-20

**Authors:** Joshua S. Catapano, Candice L. Nguyen, Andre A. Wakim, Felipe C. Albuquerque, Andrew F. Ducruet

**Affiliations:** Department of Neurosurgery, Barrow Neurological Institute, Phoenix, AZ, United States

**Keywords:** chronic subdural hematoma, cSDH, middle meningeal artery embolization, endovascular cSDH treatment, MMA embolization

## Abstract

Chronic subdural hematoma (cSDH) is a common disease process associated with significant morbidity that occurs most often in elderly patients. Asymptomatic patients are typically treated conservatively, with surgical intervention reserved for patients with symptomatic and/or large hematomas that cause brain compression. However, conservatively managed cSDH cases frequently progress, and surgical evacuation of cSDH is associated with high rates of complication and recurrence. Recently, successful treatment of cSDH via middle meningeal artery (MMA) embolization has been reported in small case series and case reports. This article reviews the existing literature on MMA embolization for cSDH and discusses the need for randomized control trials and/or large prospective studies to establish the efficacy of MMA embolization for this disease.

## Introduction

Chronic subdural hematoma (cSDH) is one of the most common neurosurgical pathologies that largely affects elderly patients and is associated with significant morbidity and mortality (1–3). cSDH is thought to evolve from a prior traumatic acute subdural hemorrhage that develops between the dura and arachnoid layer (2, 4, 5). Although this acute hematoma may resolve completely, in many cases the processes of inflammation, fibrinolysis, and/or angiogenesis lead to formation of a vascularized neomembrane that results in fluid exudation and subsequent hemorrhage, ultimately leading to volume expansion and neurological deficits (2, 4, 6). Histologically, the outer neomembrane is composed of friable sinusoidal neovessels that easily rupture spontaneously, which causes recurring hemorrhage (7–9). These neovessels derive their blood supply from the middle meningeal artery (MMA), which transverses the dura to connect to these fragile vessels (7, 8). Evidence in support of this theory comes from imaging studies reporting ipsilateral enlargement of the MMA in patients with cSDH (2, 8).

For symptomatic, refractory, and/or large cSDH causing brain compression, surgical evacuation and placement of a drain are commonly performed (10). On the other hand, asymptomatic and/or small cSDHs without brain compression are typically treated conservatively and followed-up closely with serial imaging. Successful MMA embolization for cSDH has been described in small case reports and series over the past several years (11–18). More recently, Ng et al. (19) in 2020 conducted a randomized controlled trial comparing surgery with and without MMA embolization in patients with cSDH. The authors reported one recurrence in each group. However, patients who underwent MMA had a higher hematoma reabsorption rate than those with surgical treatment alone (mean difference, 17.5 mL; 95% CI, 3.87–31.16 mL; *p* = 0.02). Embolization of the MMA is hypothesized to occlude the subdural membrane neovessels to inhibit the recurrent rupture of these vessels, thus facilitating reabsorption of the accumulated subdural fluid (20). Postembolization CT suggests that both particles and liquid embolisate can penetrate into the neomembranes, further strengthening this theory (20). Moreover, several systematic reviews and a meta-analysis have shown promising results with MMA embolization, including low rates of complication and recurrence (21, 22).

This review will discuss MMA embolization for cSDHs, focusing on the major literature studies and future directions needed to establish the efficacy of this technique as a stand-alone and/or adjunct alternative to surgical drainage.

## Discussion

### Demographic Characteristics and Treatment

cSDH typically occurs in elderly patients with or without a recognized preceding traumatic event and can be associated with the use of anticoagulant or antiplatelet medications (1–3, 10, 23–25). The occurrence of cSDHs has been steadily increasing, and with an ever-increasing elderly population, 60,000 new cases are projected each year over the next 10 years (1–3, 12, 24). Clinical manifestations of cSDH are protean; therefore, the diagnosis should be considered in any elderly patient with an altered mental status or new neurological deficit (10).

Conservative management is often reserved for asymptomatic patients without significant brain compression or midline shift (10). Such management includes frequent follow-up imaging, reversal and discontinuation of anticoagulation, and often the administration of corticosteroids (10, 26–34). Steroids are thought to inhibit the formation of the neomembrane and neovessels by suppressing both fibrinolysis and inflammation (35). However, treatment of cSDHs with corticosteroids has yielded mixed results. One systematic review found that 83–97% of patients treated with corticosteroids alone returned to neurological baseline, and only 4–27.8% would need further treatment, defined as a second round of steroids or surgical evacuation (36). A larger meta-analysis of more than 30,000 patients that included both observational studies and randomized control trials found that higher morbidity was associated with the use of corticosteroids to treat cSDHs, with no therapeutic benefit (37). Finally, a small recent randomized control trial comparing corticosteroids to placebo among patients with cSDH found that steroids may decrease the risk of requiring surgical evacuation (38). Larger randomized clinical trials are underway to investigate this therapy (39–41).

Statins are another class of medication that may be considered as conservative treatment of cSDH. This class of medication has demonstrated efficacy in reducing inflammation at the endothelium, which can also help prevent neomembrane formation (42). In addition, the role of statins in enhancing the functionality of endothelial progenitor cells (43) results in vascular protection within the brain (44). One retrospective study observed patients who were found to have a cSDH without an indication for surgery for 3 months. Chan et al. (45) found that those who were receiving atorvastatin had a 16.7% chance of experiencing deteriorating mental status, requiring bur hole drainage, compared with 58.3% of those who were not receiving atorvastatin. Finally, a recent clinical trial that included 200 patients demonstrated that among patients who had cSDH and no indication for surgery, 8 weeks of atorvastatin was associated with a significantly greater reduction in hematoma volume and improvement in neurological outcome, compared with patients who were treated without a statin. Additionally, 11.2% of those receiving atorvastatin required surgical evacuation during the study, compared with 23.5% who were not receiving a statin (46).

For patients with cSDH causing symptoms, significant midline shift, and/or neurological deficits, surgical intervention is often performed via twist drill craniotomy, bur hole craniostomy, or standard or mini craniotomy (10). Although surgical interventions are typically successful and result in clinical improvement in most patients, surgical mortality rates as high as 32% have been reported (4, 10, 24, 47, 48). Furthermore, complications and recurrence following surgical intervention occur in up to 9% and 28% of patients, respectively (10). Although the use of a subdural drain following evacuation has been reported to decrease the risk of recurrence by up to 50%, the rates of complications, recurrences, morbidity, and mortality still remain substantial (10, 49). Recently, Soleman et al. (50) conducted a randomized, controlled trial that found that subperiosteal drains are associated with an 8% rate of recurrence following bur hole drainage and lower rates of iatrogenic morbidity than subdural drain placement. Similarly, Santarius et al. (51) in 2009 conducted a randomized control trial on the use of drains vs. no drain after bur hole evacuation of cSDHs and found a low rate of recurrence of 9% among patients with a drain vs. 24% among those without a drain. Nonetheless, given the substantial risk of recurrence reported across the various surgical techniques, alternative treatment modalities, either in isolation or in combination with surgical intervention, are warranted.

**Figure 1:**
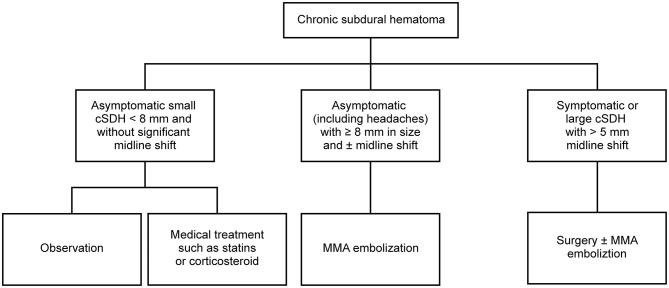
Proposed treatment algorithm for chronic subdural hematoma (cSDH) with the use of middle meningeal artery (MMA) embolization. Used with permission from Barrow Neurological Institute, Phoenix, Arizona.

MMA embolization has been shown to be effective, particularly in patients with recurrent cSDH or patients for whom surgical intervention is associated with a particularly high risk (e.g., due to receipt of anticoagulation medication or advanced comorbid conditions), and may significantly reduce the morbidity and mortality associated with cSDH; Table 1 (11, 16, 18, 22, 52–55) illustrates the major published case series in the current literature (11–18, 22, 52–55). Figure 1 shows a proposed treatment algorithm for cSDHs with the use of MMA embolization. However, level 1 evidence for the use of MMA embolization is lacking, with the majority of literature consisting of case reports or small case series.

**Table 1:** Major published studies of middle meningeal artery (MMA) embolization for chronic subdural hematoma (cSDH).

**Study**	**Year**	**Study type**	**No. of patients**	**Embolysate used**	**Major findings**
Ishihara et al. (52)	2007	Case series	7	NBCA	One of the earliest studies in which patients with cSDH were treated with MMA embolization following either a third recurrence or after a second recurrence and major bleeding risk. No recurrences were found in these patients at last follow-up (up to 15 months).
Kim (53)	2017	Prospective cohort	20	PVA	Analyzed 20 patients with recurrent cSDH treated with MMA embolization and found 85% to have a mRS <2 on follow-up; however, found no difference in mRS scores when comparing patients with traditional treatment for recurrent cSDH. Also found a significant increase in brain re-expansion, decreased hematoma recurrence, and similar complication rates to the conventional treatment group.
Ban et al. (11)	2018	Prospective cohort	72	PVA	Largest study to date; reported no complications associated with MMA embolization and found a statistically lower treatment failure rate (1.4%) than 469 conventionally treated patients (28% failure rate).
Farkas et al. (54)	2018	Case series	10	NR	Follow-up CT at 128 days showed a 45% reduction in cSDH size following MMA embolization in 10 patients (5 primary treatment vs. 5 for recurrent treatment).
Matsumoto et al. (18)	2018	Case series	4	NBCA	Four patients underwent combined MMA embolization and bur hole drainage for refractor cSDH, and none required an additional treatment vs. 2 of 10 patients who required another intervention following conventional therapy for a refractory cSDH.
Link et al. (16)	2019	Case series	49	PVA	Total of 60 SDHs embolized in 49 patients (42 primary therapy, 8 recurrences, and 10 prophylaxis prior to surgery). Reported a 91% long-term success rate, measured as either stable or decreased size of cSDH and avoidance of additional surgery.
Waquas et al. (22)	2019	Case series	8	Onyx	Six procedures were for primary therapy and two were after surgical recurrence. No retreatments recorded after embolization with complete resolution in 3 patients. All patients had a mRS <2 with an average follow-up of 3 months.
Okuma et al. (55)	2019	Case series	17	NBCA and microspheres	Nine of 17 patients with mRS ≤2 with average follow-up of 26 months.

*CT, computed tomography; mRS, modified Rankin Scale; NBCA, n-butyl cyanoacrylate; NR, not reported; PVA, polyvinyl alcohol*.

### MMA Embolization Techniques

Descriptions of the technique of MMA embolization remain scarce, with the majority of existing literature reporting the injection of microparticles (11, 16, 17, 22, 56, 57). Some authors have discussed the potential advantages of liquid embolisate for these procedures. These potentially include the depth of penetration, larger volume of embolisate, quicker procedures, increased durability, and improved visualization of the embolic agent (12). To date, no comparative studies have been performed, and further research is warranted to determine the most effective embolic agent for this procedure.

### Complications

The reported complication rate among patients who undergo MMA embolization for cSDH is low (12). The two largest series, those of Link et al. (16) (*N* = 49 patients) and Ban et al. (11) (*N* = 72 patients), reported no procedural complications attributable to MMA embolization. The recently published meta-analysis by Srivatsan et al. (21) analyzed three different double-arm studies comparing embolization and conventional surgery. The authors found a complication rate of 2% in the embolization group vs. 4% in the conventional surgical group (21).

### Recurrences

With a reported recurrence rate as high as 28% among patients with surgically treated cSDH, one of the main outcome measures analyzed in MMA embolization series is recurrence of the hemorrhage and the need for subsequent intervention. Along these lines, Link et al. (16) found that 9% of a series of 49 patients with 60 cSDHs undergoing MMA embolization required further intervention. This series included 42 hemorrhages treated for the first time, 8 postsurgical recurrences of hemorrhage, and 10 hemorrhages that occurred before surgery (16). In the largest study to date, Ban et al. (11) analyzed 72 patients with cSDH who underwent MMA embolization, including 27 asymptomatic patients who underwent embolization as sole treatment and 45 previously symptomatic patients who had previously undergone hematoma evacuation for symptomatic relief. Ban et al. (11) found a treatment failure rate (remaining or reaccumulated hematoma >1 cm in diameter at 6-month follow-up and/or the need for additional surgical procedures) of only 1.4%. Furthermore, the same authors found the treatment failure rate among 469 conventionally treated patients (402 with initial surgical evacuation and 67 with conservative management) to be significantly higher at 28% (11). Similarly, in the meta-analysis by Srivatsan et al. (21) the authors found a significantly higher hematoma recurrence rate among conventionally treated patients (28%) relative to those undergoing embolization.

### Neurological Outcomes

Although the majority of series of patients who underwent MMA embolization used the primary end point of resolution of the hematoma on follow-up imaging, four studies also reported neurological outcomes. In a series of 8 patients, Waqas et al. (22) observed that all patients had a modified Rankin Scale (mRS) score of <2 following a minimum 2-month follow-up (mean follow-up, 3 months). Similarly, Matsumoto et al. (18) published a small series of four patients with refractory cSDH who were treated with MMA embolization and found that all patients exhibited a follow-up mRS of 0. Meanwhile, Okuma et al. (55) reported 9 of 17 patients with an mRS score of ≤2 after long-term follow-up of an average of 26 months (only 3 of 17 patients had an mRS score ≤2 at admission). Lastly, Kim et al. (53) found that 17 of 20 (85%) patients with a recurrent cSDH treated with MMA embolization had an mRS <2 on follow-up. However, in the same study, 20 of 23 (87%) patients with conventional treatment exhibited similarly low mRS scores (53). Likewise, in the meta-analysis by Srivatsan et al. (21), no significant difference was found between mRS scores among patients treated with embolization embolized vs. patient who received conventional treatment.

### Future Direction

Several randomized control trials investigating the efficacy, safety, and utility of MMA embolization for cSDHs are underway (15, 16, 58–63). Additionally, various embolisates for MMA embolization are currently being studied. The SQUID Trial for the Embolization of the Middle Meningeal Artery for Treatment of Chronic Subdural Hematoma (STEM) is a randomized control trial that is investigating the safety and efficacy of SQUID for the management of cSDHS (61). Another embolisate currently being analyzed is Onyx, which is being evaluated in the Embolization of the Middle Meningeal Artery with ONYX Liquid Embolic System for Subacute and Chronic Subdural Hematoma (EMBOLISE) (62). Both of these trials are comparing medical management alone to MMA embolization, and surgical treatment with embolization to surgical treatment alone. Because the literature on MMA embolization of cSDHs includes a large number of patients who also received surgical intervention, randomized control trials will need to be conducted in a manner to also elucidate the appropriate patient selection for either MMA embolization alone or in combination with surgical intervention.

## Conclusion

MMA embolization for cSDH represents an emerging treatment modality, with a rapidly increasing number of studies analyzing this innovative and largely successful technique. However, many questions remain, including appropriate patient selection, efficacy as a stand-alone procedure, optimal embolization techniques, and timing of embolization with regard to surgical intervention in symptomatic patients. Furthermore, the majority of literature on MMA embolization for cSDH reports cases studies and small case series, whereas several randomized control trials have shown efficacy with surgical interventions. Hence, many randomized clinical trials using MMA as a treatment for cSDH are underway at several centers in the United States and Europe (58–65). These studies will ultimately provide insight on the safety, efficacy, and use of this novel technique in the treatment of cSDHS.

## Author Contributions

JC: manuscript writing, editing, and literature review. CN and AW: manuscript writing and literature review. FA: editing. AD: editing and final approval. All authors contributed to the article and approved the submitted version.

## Conflict of Interest

AD is a consultant for Medtronic, Stryker, Cerenovus, Penumbra, and Koswire. The remaining authors declare that the research was conducted in the absence of any commercial or financial relationships that could be construed as a potential conflict of interest.

## Abbreviations

cSDH: chronic subdural hematoma
MMA: middle meningeal artery
mRS: modified Rankin Scale.
