# Corrigendum: Middle Meningeal Artery Embolization for Chronic Subdural Hematoma

**DOI:** 10.3389/fneur.2021.666701

**Published:** 2021-03-22

**Authors:** Joshua S. Catapano, Candice L. Nguyen, Andre A. Wakim, Felipe C. Albuquerque, Andrew F. Ducruet

**Affiliations:** Department of Neurosurgery, Barrow Neurological Institute, Phoenix, AZ, United States

**Keywords:** chronic subdural hematoma, cSDH, middle meningeal artery embolization, endovascular cSDH treatment, MMA embolization

In the original article, there were errors in the text.

A correction has been made to *Discussion, Future Direction*. The corrected paragraph is shown below:

Several randomized control trials investigating the efficacy, safety, and utility of MMA embolization for cSDHs are underway (15, 16, 58–63). Additionally, various embolisates for MMA embolization are currently being studied. The SQUID Trial for the Embolization of the Middle Meningeal Artery for Treatment of Chronic Subdural Hematoma (STEM) is a randomized control trial that is investigating the safety and efficacy of SQUID for the management of cSDHS (61). Another embolisate currently being analyzed is Onyx, which is being evaluated in the Embolization of the Middle Meningeal Artery with ONYX Liquid Embolic System for Subacute and Chronic Subdural Hematoma (EMBOLISE) (62). Both of these trials are comparing medical management alone to MMA embolization, and surgical treatment with embolization to surgical treatment alone. Because the literature on MMA embolization of cSDHs includes a large number of patients who also received surgical intervention, randomized control trials will need to be conducted in a manner to also elucidate the appropriate patient selection for either MMA embolization alone or in combination with surgical intervention.

The authors apologize for this error and state that this does not change the scientific conclusions of the article in any way. The original article has been updated.

